# The contribution of photosynthesis traits and plant height components to plant height in wheat at the individual quantitative trait locus level

**DOI:** 10.1038/s41598-020-69138-0

**Published:** 2020-07-23

**Authors:** Ma Yu, Ze-Hou Liu, Bin Yang, Hua Chen, Hong Zhang, Da-Bin Hou

**Affiliations:** 1 School of Life Science and Engineering, Southwest University of Science and Technology, 59 Qinglong Road, Mianyang, 621010 Sichuan China; 2 Crop Research Institute, Sichuan Academy of Agricultural Sciences, Chengdu, 610066 Sichuan China

**Keywords:** Genetics, Plant sciences

## Abstract

Plant height is an important agronomic trait for morphogenesis and grain yield formation in wheat. In this study, we performed both normal and multivariate conditional quantitative trait locus (QTL) analyses for plant height with spike length, internode number, length of the first internode to the sixth internode from the top during harvest, and photosynthesis traits at the seedling stage and heading stage based on a recombinant inbred line population. A total of 49 normal QTLs were detected, as well as 312 conditional QTLs. The genetic region *Xbcd1970*-*Xbcd262* on chromosome 2D harbored the most QTLs, with 6 normal QTLs and 39 conditional QTLs. A comparison between the normal and conditional QTL mapping analyses suggested that the length of the third internode, fourth internode, and fifth internode from the top showed a high genetic association with plant height, whereas all photosynthesis traits showed weaker associations. This comparative analysis could serve as a platform for dissecting the genetic relation between objective traits and other phenotypic traits before manipulation of genes collocated with QTL clusters.

## Introduction

Plant height is an important factor in terms of morphogenesis and grain yield of wheat. An appropriate plant height is associated with a reduced incidence of lodging, an increased grain number per spike, and an improved harvest index and thus increased grain yield and quality^1,2^. Gaudet and colleagues hypothesized that reduced culm height was associated with bunt susceptibility^3^. In addition, Mao and coauthors demonstrated a negative association between plant height and Fusarium head blight resistance via a quantitative trait locus (QTL) meta-analysis of the results of fifty-six previous experiments^4^. Therefore, understanding the association of different traits with plant height has the potential to assist breeders in more efficiently selecting related traits.

Several studies have been conducted to unravel the genetic control of plant height in wheat. To date, 25 *Reduced height* (*Rht*) genes have been identified across chromosomes 2A, 2B, 2D, 3B, 4A, 4B, 4D, 5A, 5D, 6A, 7A, and 7B in wheat^5,6^. The introduction of these semidominant dwarfing alleles has reduced the plant height of modern wheat cultivars. In addition to these major *Rht* genes, the vernalization requirement gene *Vrn-A1*, photoperiod gene *Ppd-D1*, and a number of major QTLs for plant height have also been reported in wheat^7–9^.

Plants can convert sunlight energy into biomass via photosynthesis, the theoretical maximum conversion efficiency of which is 12%^10^. Previous studies have focused on increasing this photosynthetic efficiency by using single genes during plant selection and breeding^11,12^. However, insertion of single genes can result in changes to many other traits, such as physiological processes and morphological structures. Thus far, efforts to improve photosynthetic efficiency have proven unsuccessful^13^, and optimizing photosynthetic systems likely requires the dissection of the genetic relationships between engineered systems and other traits.

Conditional analysis has been widely used in several crop species to quantify the respective contributions of both component traits and interrelated traits to more complex target traits^14–17^. In the present study, we used a recombinant inbred line (RIL) population to design a QTL mapping analysis of plant height and its component traits, including spike length, length of the first internode to the sixth internode from the top, and internode number. Additionally, we also performed a conditional analysis to quantify the genetic relationship between plant height and plant height components, photosynthesis traits, and transpiration rate.

## Material and methods

### Plant materials and experimental design

The experimental design used in the present study has been described previously^17^. Briefly, the International Triticeae Mapping Initiative (ITMI) population comprising 112 RILs was used^18^. This population was derived from the synthetic hexaploid wheat cultivar W7984 and the winter wheat cultivar Opata 85. The two parents and all RILs were grown in Mianyang, Sichuan Province (31°32′ N and 104°42′ W), from 2015 to 2017. Each line was planted in five 1.5 m rows. The distance between two rows was 30 cm, and the seeding rate was 10 cm per plant. Three repetitions were included in accordance with a randomized complete block design in each year.

### Phenotypic evaluations

For each line, all phenotypic traits were evaluated using five representative leading tillers. Plant height and its component traits, including spike length, internode number, and the length of the first internode to the sixth internode from the top, were measured during harvest.

Five photosynthesis traits, chlorophyll content, the net photosynthetic rate, intercellular CO_2_ concentration, the transpiration rate, and stomatal conductance, were investigated at both the seedling and heading stages. The chlorophyll content was measured using a SPAD-502 Plus chlorophyll meter (Minolta, Osaka, Japan), and the other four photosynthesis traits were estimated using an LI-6400XT Portable Photosynthesis system (LI-COR, Lincoln, USA). Data from two consecutive years (E1 of 2015–2016 and E2 of 2016–2017) were combined and then used as phenotypic traits in E3.

### Statistical analysis

Analyses of variance and analysis of covariance (ANCOVA) were based on the minimum norm quadratic unbiased estimation proposed by Zhu^19^. The phenotypic and genetic correlations among all the traits were investigated via the multivariate restricted maximum likelihood (REML) by PROC MIXED of SAS software version 9.4^20,21^. A network analysis was carried out with the R package ‘qgraph’^22,23^, and heritability was estimated following the approach suggested by Ma et al.^24^. Conditional analysis was performed using the QGAStation software package 1.0^25^, employing the ‘QTLData’ menu settings as described previously^15^. The output file of QGAStation provides the conditional phenotypic values *y*_(A|B)_, which indicate a new value of A without the influence of B. In the conditional analysis, plant height was considered the final complex trait, whereas the plant height components and photosynthesis traits were considered causal traits.

### Genetic mapping and QTL analysis

The genetic linkage map of the ITMI population used in our study was generated by Song and coauthors^26^, was 2541 cM in size, contained 1,410 distinct loci and covered 2,541 cM of genetic distance. Inclusive composite interval mapping was performed using IciMapping 4.1^27^. The walking speed and LOD threshold parameters were as previously described^17^. Both the normal phenotypic values and the new conditional phenotypic values were used in the QTL mapping analyses. These analyses were initially performed separately for each year and then carried out using data combined across both years.

### Conditional analysis at the QTL level

The comparative analysis of normal and conditional QTL mapping had four possible outcomes: (1) a normal QTL for trait A with no identification for the conditional trait of y(A|B), suggesting that this QTL for trait A is derived fully from trait B; (2) a normal QTL for trait A can also be detected for y(A|B) with a phenotypic variation range greater than 10%, indicating that this QTL for trait A is partly affected by trait B; (3) a normal QTL for trait A was also detected for y(A|B) with similar phenotypic variation, indicating that this QTL for trait A is totally independent of trait B; (4) only one additional QTL for y(A|B) was identified, suggesting that the expression of the QTL for trait A is suppressed by trait B, and this expression could be identified by elimination of trait B influence.

## Results

### Phenotypic evaluations

The data for all phenotypic traits assessed here are presented in Table 1. On average, the synthetic hexaploid parent of W7984 has more than six internodes, whereas Opata 85 has only five internodes.

**Table 1:** Phenotypic summary and heritability of traits investigate for population lines and parent lines.

Traits^a^	Environment	Parents		Population			
		Opata85	W7984	Mean	SD	Range	Heritability (%)
PH (cm)	2015–2016	101.82	118.98	117.54	11.09	88.13–142.70	76.10
	2016–2017	98.20	120.45	117.34	11.31	90.70–142.20	
	Overall	97.10	119.17	117.36	11.18	88.92–142.25	
SL (cm)	2015–2016	10.41	10.00	10.76	1.52	7.15–15.27	71.46
	2016–2017	9.40	10.15	10.80	1.64	7.45–15.15	
	Overall	9.93	10.39	10.78	1.56	7.35–15.15	
1st (cm)	2015–2016	40.96	37.50	40.20	4.50	27.24–51.42	60.33
	2016–2017	40.80	38.00	40.30	4.80	24.05–54.40	
	Overall	40.69	37.45	40.25	4.59	25.59–52.91	
2nd (cm)	2015–2016	19.86	19.73	22.27	3.30	13.27–29.33	74.76
	2016–2017	19.85	22.00	22.28	3.39	12.7–29.75	
	Overall	19.86	20.63	22.27	3.32	12.98–29.34	
3rd (cm)	2015–2016	13.40	15.43	15.92	2.35	10.67–22.53	71.60
	2016–2017	13.00	15.75	16.04	2.52	10.95–24.75	
	Overall	13.29	15.80	15.98	2.41	10.83–23.025	
4th (cm)	2015–2016	9.98	14.00	12.59	1.84	8.38–18.4	71.90
	2016–2017	9.50	14.00	12.59	1.79	9.00–18.15	
	Overall	9.84	13.47	12.59	1.79	8.80–18.275	
5th (cm)	2015–2016	6.80	11.63	9.89	2.24	3.90–15.28	53.47
	2016–2017	5.65	12.00	9.81	2.34	4.45–15.70	
	Overall	6.47	10.86	9.85	2.24	4.18–15.49	
6th (cm)	2015–2016	0.00	8.90	5.12	3.01	0.00–12.33	69.12
	2016–2017	0.00	8.55	4.74	3.42	0.00–12.1	
	Overall	0.00	8.92	4.93	3.15	0.00–12.17	
IN	2015–2016	5.00	6.25	5.85	0.56	4.75–7.67	57.73
	2016–2017	5.00	6.00	5.83	0.61	4.50–7.50	
	Overall	5.00	6.17	5.84	0.58	4.63–7.58	
CNS (SPAD value)	2015–2016	37.20	48.20	44.46	3.35	36.30–54.60	61.38
	2016–2017	42.40	51.30	41.50	3.69	31.10–52.80	
	Overall	39.50	49.60	42.98	2.86	37.50–53.45	
CNH (SPAD value)	2015–2016	43.39	47.40	44.55	5.15	24.30–52.80	77.83
	2016–2017	48.50	50.14	45.93	4.11	35.70–56.00	
	Overall	45.10	49.00	45.01	4.51	29.90–54.70	
PNS (μmol s^−1^ m^−2^)	2015–2016	13.72	14.48	13.14	4.69	4.97–24.36	74.71
	2016–2017	15.54	16.47	14.49	2.42	8.44–21.5	
	Overall	14.32	15.14	13.88	3.22	7.63–21.36	
PNH (μmol s^−1^ m^−2^)	2015–2016	17.49	17.42	14.98	2.36	9.63–20.16	81.89
	2016–2017	17.66	17.77	14.93	3.17	8.21–20.54	
	Overall	17.55	17.52	14.96	3.00	9.09–20.17	
GsS (m mol s^−1^ m^−2^)	2015–2016	0.27	0.52	0.37	0.12	0.12–0.87	56.71
	2016–2017	0.31	0.56	0.38	0.14	0.11–0.81	
	Overall	0.30	0.55	0.38	0.10	0.12–0.72	
GsH (m mol s^−1^ m^−2^)	2015–2016	0.57	0.48	0.54	0.12	0.22–0.78	50.40
	2016–2017	0.41	0.31	0.36	0.09	0.11–0.57	
	Overall	0.50	0.41	0.50	0.10	0.13–0.70	
CiS (mol L^-1^)	2015–2016	239.64	341.97	282.17	54.94	129.79–374.58	42.21
	2016–2017	282.83	377.76	307.61	85.94	153.39–407.78	
	Overall	250.59	358.53	296.39	54.99	181.23–418.5	
CiH (mol L^-1^)	2015–2016	305.82	299.61	315.78	18.64	246.27–363.89	36.04
	2016–2017	275.81	270.83	281.93	19.51	217.84–332.57	
	Overall	299.57	286.56	300.78	20.22	258.00–331.57	
TRS (m mol s^−1^ m^−2^)	2015–2016	2.89	4.87	3.35	1.57	0.81–8.66	44.3
	2016–2017	3.14	5.29	3.49	1.21	1.07–7.41	
	Overall	3.09	4.96	3.44	1.05	1.59–6.37	
TRH (m mol s^−1^ m^−2^)	2015–2016	4.78	5.88	4.66	1.14	2.18–8.28	57.52
	2016–2017	4.43	6.18	4.36	0.90	2.34–7.05	
	Overall	4.63	5.99	4.56	1.02	2.66–7.55	

^a^Traits are plant height (PH); spike length (SL); internode number (IN); length of first internode from the top (1st); length of second internode from the top (2nd); length of third internode from the top (3rd); length of fourth internode from the top (4th); length of fifth internode from the top (5th); length of sixth internode from the top (6th); chlorophyll content at seedling stage (CNS) and heading stage (CNH); net photosynthetic rate at seedling stage (PNS) and heading stage (PNH); stomatal conductance at seedling stage (GsS) and heading stage (GsH); intercellular CO2 concentration at seedling stage (CiS) and heading stage (CiH); transpiration rate at seedling stage (TRS) and heading stage (TRH); respectively.

We first assessed plant height and its related component traits; the W7984 parent was 22.07 cm taller on average than was the Opata 85 parent (*P* < 0.05). The internode length was 8.65% longer for the top node of Opata 85 but was 3.73%, 15.90%, 26.95%, and 40.42% longer for that of W7984 from the 2nd node to the 5th node, respectively (*P* < 0.05). W7984 had a higher chlorophyll content than Opata 85 at the seedling stage (*P* < 0.05), but the difference decreased at the heading stage. Similar trends were observed for photosynthetic capacity, and transgressive segregation was observed for all traits in the population.

**Figure 1:**
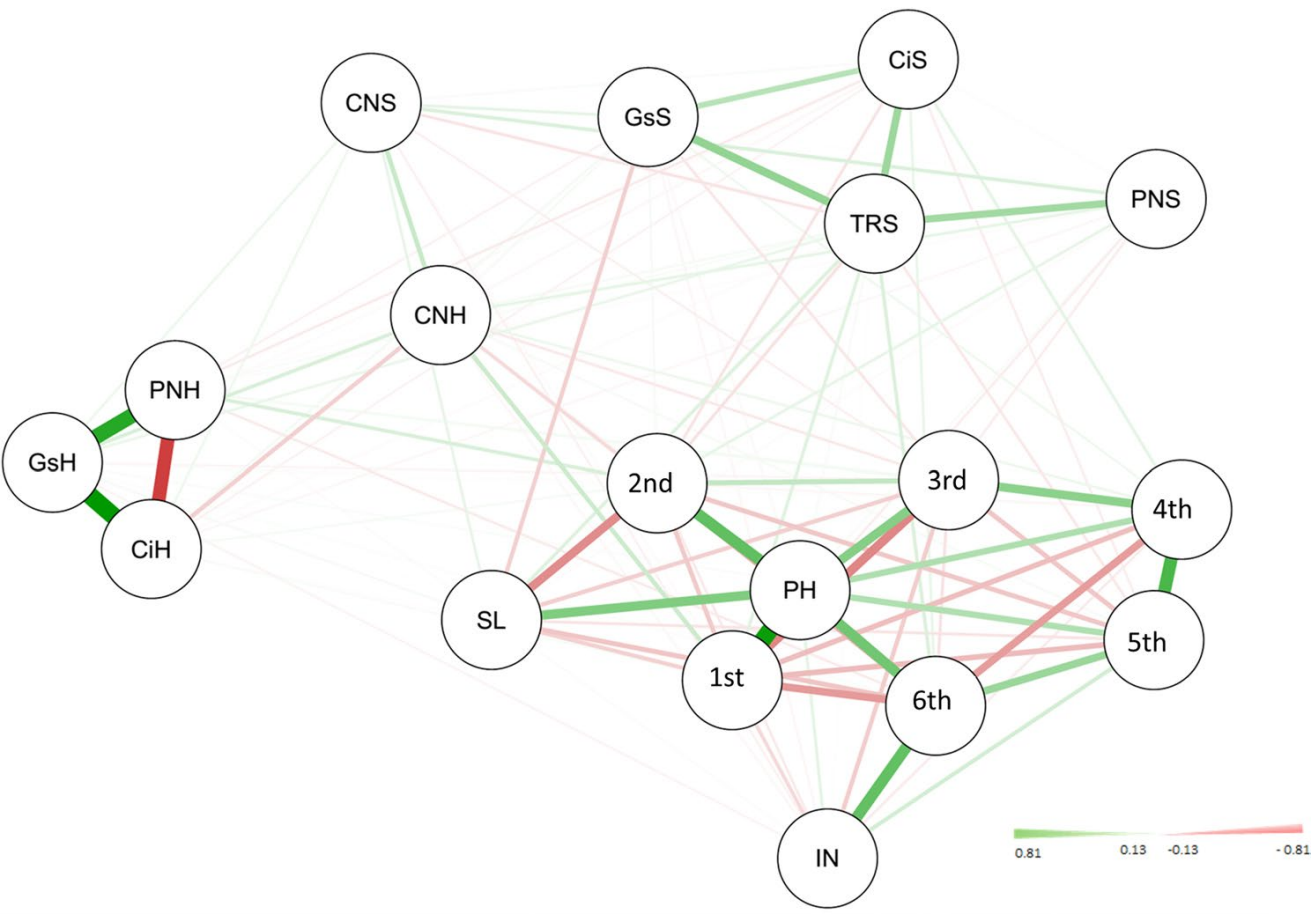
Gnetic correlations among the investigated traits and plant height. Traits are plant height (PH); spike length (SL); internode number (IN); length of first internode from the top (1st); length of second internode from the top (2nd); length of third internode from the top (3rd); length of fourth internode from the top (4th); length of fifth internode from the top (5th); length of sixth internode from the top (6th); chlorophyll content at seedling stage (CNS) and heading stage (CNH); net photosynthetic rate at seedling stage (PNS) and heading stage (PNH); stomatal conductance at seedling stage (GsS) and heading stage (GsH); intercellular CO2 concentration at seedling stage (CiS) and heading stage (CiH); transpiration rate at seedling stage (TRS) and heading stage (TRH); respectively.

The highest heritability was observed for the net photosynthetic rate at the heading stage, followed by the chlorophyll content, while the intercellular CO_2_ concentration at the heading stage exhibited the lowest heritability value of 36.04% during the two years. Genetic correlation analysis showed that plant height was positively correlated with the spike length (34%), first internode length from the top (48%), second internode length from the top (62%), third internode length from the top (69%), fourth internode length from the top (77%), fifth internode length from the top (69%), sixth internode length from the top (47%), net photosynthetic rate at the heading stage at the heading stage (21%), and intercellular CO_2_ concentration at the heading stage (20%) (Table 2, Fig. 1).

**Table 2:** Phenotypic correlation coefficient and genetic correlation coefficient between the photosynthetic and plant height related traits.

	PH	IN	SL	1st	2nd	3rd	4th	5th	6th	CNS	CNH	PNS	PNH	CiS	CiH	GsS	GsH	TRS	TRH
PH	–	NS	0.34	0.48	0.62	0.69	0.77	0.69	0.47	NS	NS	NS	0.21	NS	NS	NS	0.2	NS	NS
IN	NS	–	NS	− 0.24	− 0.29	NS	NS	0.65	0.87	NS	− 0.36	NS	− 0.3	0.38	NS	NS	− 0.32	− 0.21	0.35
SL	0.33	NS	–	0.22	NS	NS	NS	NS	NS	NS	NS	NS	NS	NS	NS	NS	NS	0.22	0.29
1st	0.47	− 0.24	0.2	–	0.35	NS	NS	NS	NS	NS	0.34	NS	NS	NS	NS	NS	0.27	0.21	NS
2nd	0.61	− 0.28	NS	0.34	–	0.74	0.53	NS	NS	NS	NS	NS	0.43	− 0.34	NS	NS	0.33	− 0.34	− 0.32
3rd	0.68	NS	NS	NS	0.72	–	0.8	0.42	NS	NS	NS	NS	0.34	NS	NS	− 0.25	0.22	− 0.3	NS
4th	0.76	NS	NS	NS	0.52	0.78	–	0.75	0.28	NS	NS	NS	0.26	NS	NS	NS	0.24	NS	NS
5th	0.67	0.63	NS	NS	NS	0.41	0.74	–	0.76	− 0.26	− 0.32	NS	NS	NS	NS	NS	NS	NS	0.33
6th	0.49	0.85	NS	NS	NS	NS	0.29	0.75	–	NS	− 0.4	NS	− 0.27	0.42	NS	NS	− 0.26	− 0.26	0.48
CNS	NS	NS	NS	NS	NS	NS	NS	− 0.23	NS	–	0.39	0.27	NS	NS	NS	NS	NS	NS	NS
CNH	NS	− 0.31	NS	0.29	NS	NS	NS	− 0.27	− 0.34	0.29	–	NS	NS	− 0.23	NS	NS	0.36	NS	NS
PNS	NS	NS	NS	NS	NS	NS	NS	NS	NS	0.2	NS	–	NS	NS	NS	0.21	NS	0.42	NS
PNH	0.19	− 0.29	NS	NS	0.41	0.31	0.24	NS	− 0.26	NS	NS	NS	–	− 0.29	− 0.27	NS	0.81	NS	− 0.21
CiS	NS	0.25	NS	NS	− 0.22	NS	NS	NS	0.29	NS	− 0.07	NS	− 0.19	–	NS	0.48	NS	0.48	0.25
CiH	NS	NS	NS	NS	NS	NS	NS	NS	NS	NS	NS	NS	− 0.16	NS	–	NS	NS	NS	NS
GsS	NS	NS	NS	NS	NS	− 0.2	NS	NS	NS	NS	NS	0.19	NS	0.41	NS	–	NS	0.46	NS
GsH	0.13	− 0.22	NS	0.18	0.23	0.15	0.16	NS	− 0.17	NS	0.26	NS	0.6	NS	NS	NS	–	NS	NS
TRS	NS	− 0.14	0.14	0.14	− 0.23	− 0.2	NS	NS	− 0.17	NS	NS	0.36	NS	0.5	NS	0.49	NS	–	0.52
TRH	NS	0.26	0.21	NS	− 0.23	NS	NS	0.24	0.35	NS	NS	NS	− 0.15	NS	NS	NS	NS	0.25	–

^a^ns is insignificant r-values *p* > 0.05. Data in lower left quarter of the matrix are the simple correlation coefficients, and data in the top right corner are the genetic correlation coefficients. The full name of traits were listed in Table 1.

### QTL mapping

A total of 49 QTLs for 19 normal traits (Supplementary information Table S1), explaining between 5.20% and 27.71% of the phenotypic variance, were identified in the ITMI population. Among these, 25 QTLs were detected in at least two of the three environments, and 11 were present in all environments. The largest number of QTLs (five) were identified for stomatal conductance and chlorophyll content at the seedling stage, whereas no QTLs for intercellular CO_2_ concentration at the heading stage were found in any environment. The combination of normal and conditional QTL mapping revealed a total of 40 genetic regions clustered within two to 45 QTLs across all 21 chromosomes except for chromosomes 4B, 6D, and 7D. The genetic region of *Xbcd1970-Xbcd262* on chromosome 2D harbored the most QTLs, with 6 normal QTLs and 39 conditional QTLs, followed by *Xmwg522-Xbarc330* on 5A, with 2 normal QTLs and 33 conditional QTLs.

Four putative QTLs for plant height were identified on 1A, 5A, and 6A, and each explained between 8.95 and 17.28% of the phenotypic variance. The QTLs on 1A and 5A (*Xgwm293-Xcdo785*) were detected in E2 and the combined years of E3. The alleles of all QTLs from the W7984 parent contributed to increased plant height.

A total of nine QTLs were detected for plant height component traits (total internode number, internode length, spike length) across all environments, with between one and four identified for each. Genome A harbored the most QTLs, with 13 identified across chromosomes 2A, 3A, 4A, 5A, and 6A. Genome D had six QTLs on chromosomes 1D and 2D. Only six QTLs were identified on 2B, 4B, 5B, and 6B of the B genome. The QTL for the length of the sixth internode from the top found in the region *XBcd1970*-*XBcd262* on chromosome 2D explained the most phenotypic variation, with an average of 26.93% across all environments. The same region also contained stable QTLs for the length of the second internode from the top, the length of the fifth internode from the top, and the total internode number.

### QTL analysis for plant height conditioned upon plant height components and photosynthesis traits

When the influences of the third internode length from the top, the fourth internode length from the top, and the fifth internode length from the top on plant height were removed, no QTLs for plant height were detected in any environment (Table 3), suggesting a high genetic association between these three component traits and plant height. Similar and weaker associations were observed for the influences of the first internode length and the second internode length from the top, respectively, on plant height. At least one normal QTL for plant height was detected using conditional analysis for all photosynthesis traits, with the exception of the chlorophyll content at the seedling stage and net photosynthetic rate at both seedling and heading stages for the combined yearly data.

**Table 3:** Number of unconditional and conditional QTL for plant height detected in different environments.

Env.^a^	Uncon.^b^	Stage^c^	Con.^d^												
			PH|Ci	PH|CN	PH|Gs	PH|PN	PH|TR	PH|SL	PH|1st	PH|2nd	PH|3rd	PH|4th	PH|5th	PH|6th	PH|IN
E1	2	S	1 + 0 + 2	1 + 0 + 2	1 + 0 + 1	1 + 0 + 1	1 + 0 + 2	0 + 0 + 1	2 + 0 + 3	1 + 0 + 3	0 + 0 + 2	0 + 0 + 2	0 + 0 + 3	0 + 0 + 3	0 + 0 + 2
		H	2 + 0 + 0	2 + 0 + 0	1 + 0 + 3	2 + 0 + 0	1 + 0 + 2								
E2	2	S	1 + 0 + 3	1 + 0 + 1	2 + 0 + 1	1 + 0 + 2	1 + 0 + 2	2 + 0 + 0	0 + 0 + 1	0 + 0 + 4	0 + 0 + 2	0 + 0 + 0	0 + 0 + 3	1 + 0 + 2	2 + 0 + 1
		H	1 + 0 + 2	1 + 0 + 1	1 + 0 + 3	1 + 0 + 2	1 + 0 + 2								
E3	2	S	1 + 0 + 3	0 + 0 + 1	0 + 0 + 2	2 + 0 + 2	1 + 0 + 3	2 + 0 + 0	0 + 0 + 1	0 + 0 + 5	0 + 0 + 1	0 + 0 + 0	0 + 0 + 2	1 + 0 + 3	1 + 0 + 2
		H	1 + 0 + 2	1 + 0 + 2	0 + 0 + 2	1 + 0 + 1	1 + 0 + 2								

^a^Environment are E1: Mian’yang in 2015–2016, E2: Mian’yang in 2016–2017, E3: combined environment over the 2 years.

^b^Number of unconditional QTL for grain yield.

^c^Stage for photosynthesis traits are S: seedling stage, H: heading stage.

^d^Number of conditional QTL. The first Arabic numerals before the ‘‘ + ’’ indicate the number of QTL detected in both unconditional and conditional analyses with equal phenotypic variation (phenotypic variation of conditional QTL ranged less than 10% of that of the corresponding unconditional QTL); the middle Arabic numerals indicate the number of QTL detected in both unconditional and conditional analyses with reduced or enhanced phenotypic variation; the third Arabic numerals following a ‘‘ + ’’ indicate the number of QTL that was detected only in conditional analysis.

Conditional analysis also allowed the identification of additive QTLs not detected using traditional QTL analysis. Removing the influence of the second internode length from the top allowed the identification of the greatest number of extra QTLs contributing to plant height, with three, four, and five identified in environments E1, E2, and E3, respectively. The same approach allowed for the identification of 8 QTLs contributing to the fifth internode length from the top, the sixth internode length from the top, stomatal conductance at the heading stage, and the intercellular CO_2_ concentration at the seedling stage.

The combination of normal QTLs that disappeared and extraconditional QTLs indicated a higher genetic relationship between plant height and its components than between plant height and photosynthesis traits, with the exception of spike length and internode number. Similar tendencies were observed for the phenotypic and genetic correlation analyses.

## Discussion

### QTLs for plant height with respect to previous studies

Previous genetic studies have identified numerous major QTLs for plant height on the majority of the 21 wheat chromosomes^15^. The semidominant GA-responsive dwarfing gene *Rht18* is linked to *Xbarc3* on the short arm of chromosome 6A^28^ and is a candidate gene for inclusion in breeding programs to improve wheat performance^29^. This gene was identified in the present study between *Xcdo29* and *Xtam36*. The QTL on 1A is located in approximately the same region as *QHt.fra-1A* is^30^. A major QTL on 5A in the genetic region of *Xmwg522*-*Xbarc330* was mapped to a position where *QHt.inra-5A* was found, but it was identified only in the environment of E1. Additionally, a major QTL in the genetic region of *Xgwm293-Xcdo785* on 5A was identified in E2 and E3, confirming the findings of previous reports^31^.

### Genetic relationship between plant height and its components and photosynthesis traits at specific QTLs

**Figure 2:**
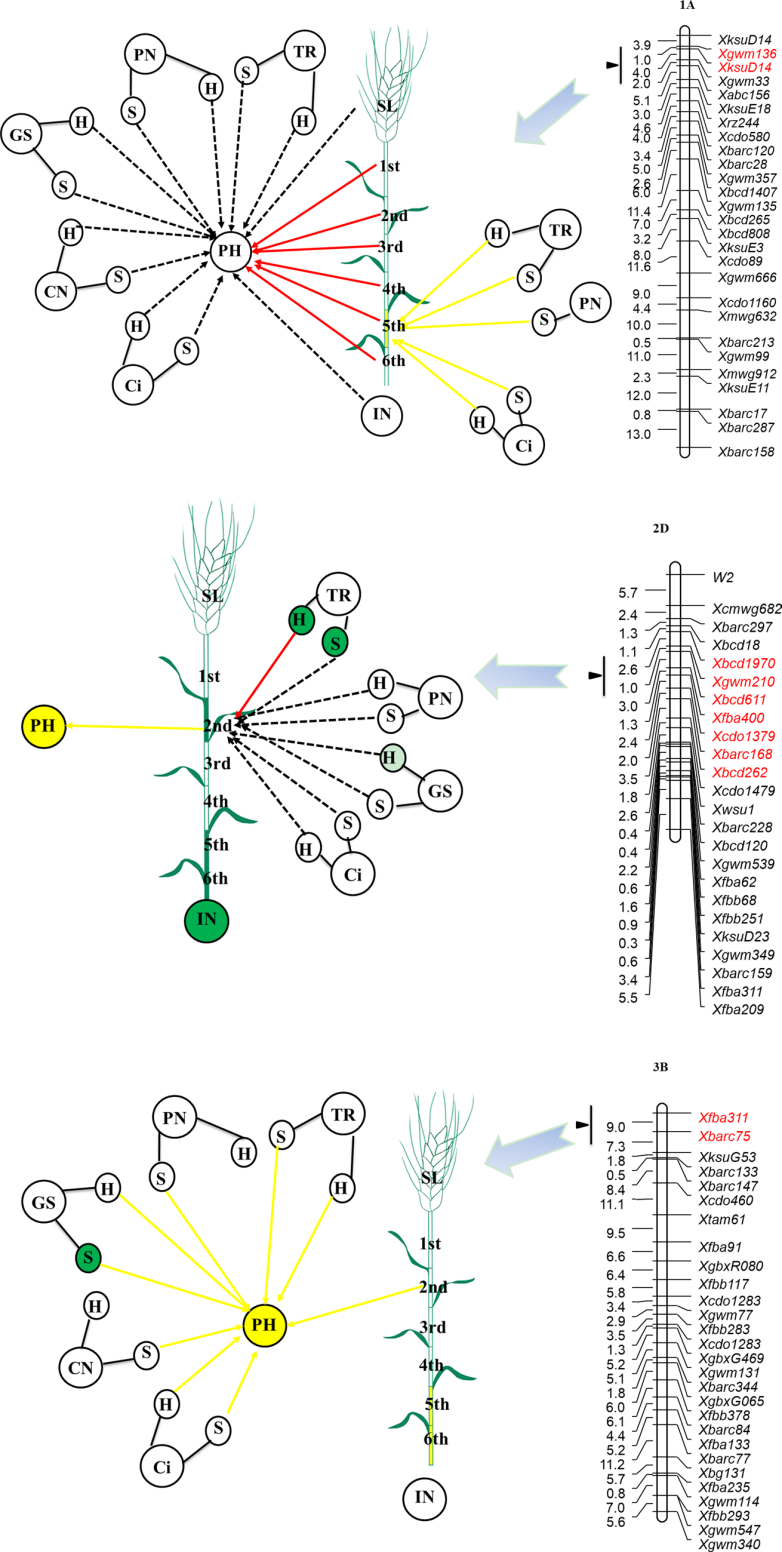

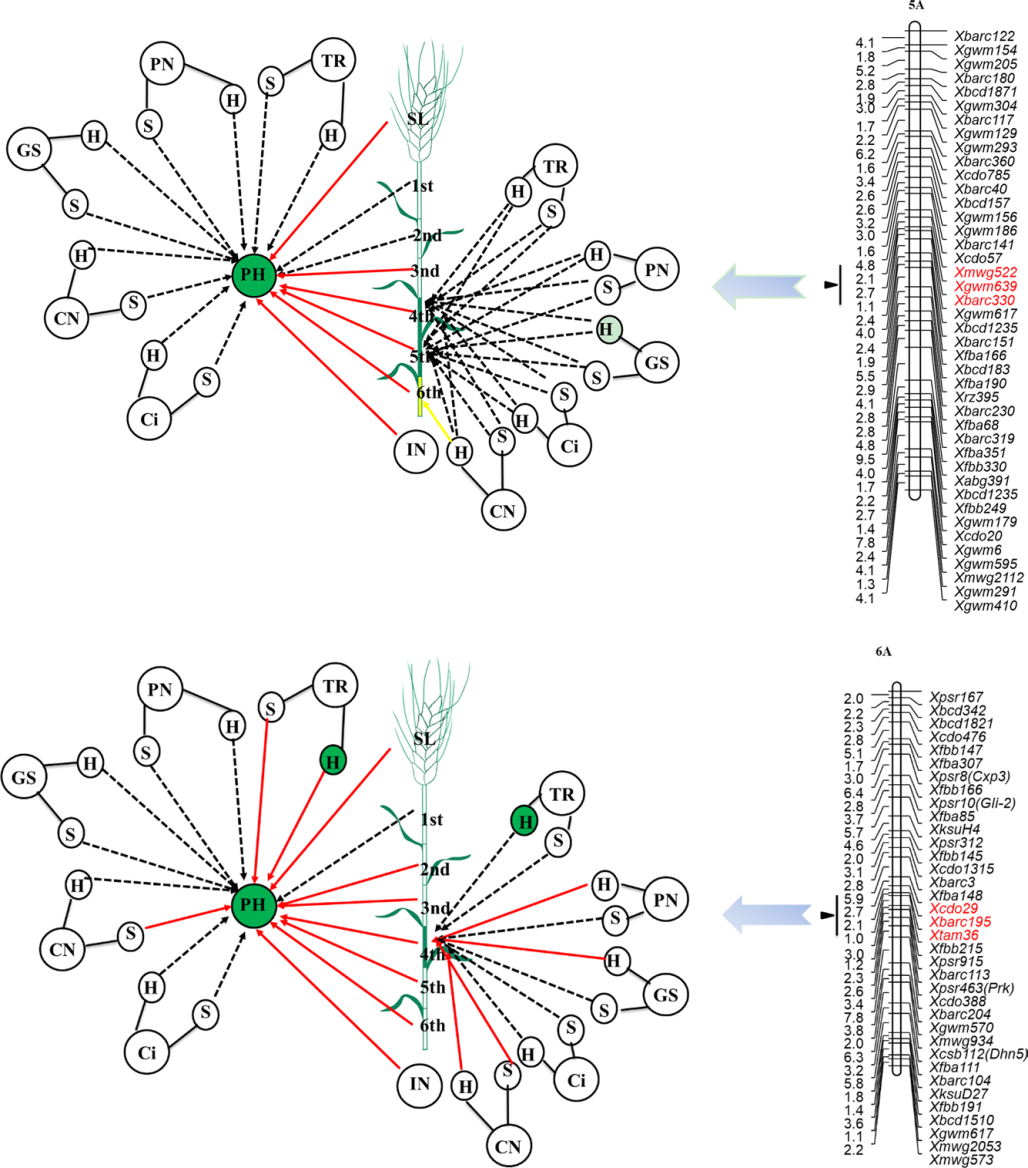
QTL clusters carried normal QTL or conditional QTL for plant height. Traits are plant height (PH); spike length (SL); internode number (IN); length of first internode from the top (1st); length of second internode from the top (2nd); length of third internode from the top (3rd); length of fourth internode from the top (4th); length of fifth internode from the top (5th); length of sixth internode from the top (6th); chlorophyll content (CN); net photosynthetic rate (PN); stomatal conductance (GS); intercellular CO2 concentration (Ci); transpiration rate (TR); respectively. S and H indicating seedling stage and heading stage respectively. The networks are comparison of conditional QTL and normal QTL analysis. Yellow arrow indicated suppression between plant height components or photosynthetic traits with plant height, or photosynthetic traits with plant height components, whereas the red arrow indicated positive contribution between two traits. Dotted arrow indicated independent relationship between two traits. Traits with green background or green internode indicated normal QTLs were identified, whereas traits with yellow background or yellow internode indicated only conditional QTLs were identified.

Comparative analyses of normal and conditional QTL mapping can dissect genetic relationships between plant height and related traits. A normal QTL for plant height was identified on 1A in the interval between *Xgwm136* and *XksuD14* (Supplementary information Table S1, Fig. 2). However, this QTL was no longer detected once the influences of the first internode length to the sixth internode length were removed. Therefore, we concluded that at this locus, variation in plant height was attributable entirely to internode length (the first to the sixth internodes). While no normal QTLs for plant height components were detected at this locus, conditional QTLs for the fifth internode length were identified when the influence of intercellular CO_2_ concentration at the seedling and heading stages, the net photosynthetic rate at the seedling stage, and the transpiration rate at both stages were removed. This indicated that the fifth internode length was suppressed by these photosynthesis traits. However, the relationship between these photosynthesis traits and plant height was nonsignificant during the direct conditional analysis.

For the locus found at *Xmwg522*-*Xbarc330* on 5A, plant height was entirely attributable to the variation in spike length, internode number, and the third internode length to the sixth internode length. We also identified a normal QTL for the fourth internode length and the fifth internode length, but their genetic correlations with photosynthesis traits were insignificant. A conditional QTL was also identified at this location, but its expression was suppressed by the chlorophyll content at the heading stage. Previous work has described two single-nucleotide polymorphism (SNP) markers (IWA6949 and IWA6573) that are associated with the MIKC-type MADS-box gene(s) of WM10A (TraesCS5A02G293000 in the Ensembl plant database) in the same genomic region^32,33^. MIKC-type MADS-box genes are involved in virtually all aspects of plant development in a variety of important crop species, including wheat, rice, and banana^34–36^. Manipulation of this gene might be an avenue to improve important agronomic traits, such as plant height.

For the locus in the region of *Xcdo29-Xtam36* on 6A, plant height was entirely controlled by the chlorophyll content at the seedling stage, the transpiration rate at the seedling stage, and all plant height components, with the exception of the first internode length. A normal QTL for the fourth internode length was identified at this locus. Its expression entirely contributed to chlorophyll content at the seedling stage and heading stage and both stomatal conductance and net photosynthetic rate at the heading stage. The contribution of normal QTLs to the transpiration rate at the heading stage to the fourth internode length was insignificant. Previous studies have shown that the *Xcdo29-Xtam36* region contains a SNP marker (IWA1520) associated with the *WAVE-DAMPENED 2-LIKE* gene of TraesCS6A02G182000 in the Ensembl plant database^32,33^. High constitutive expression of this gene caused short and thick stems in *Arabidopsis thaliana*^37^.

### Utilization of conditional analysis for plant breeding and pathway network construction

In plant breeding, manipulation of a single gene typically results in changes to several phenotypic traits; for example, overexpression of H^+^-ATPase results in enhanced light-induced stomatal opening, photosynthesis, and plant growth in *Arabidopsis*^38^. Engineering plants to carry favorable traits such as those that promote disease resistance, hardiness, and yield quality can also result in the manifestation of unwanted characteristics such as excessive height, late heading and maturity, hardened glumes, and increased threshing difficulty^39^. Thus, the improvement of a single trait requires examination of the genetic relationships between the target trait and other phenotypic traits prior to gene manipulation. Taking the first internode length as an example, this trait previously showed a positive correlation with lodging resistance and kernel weight^40,41^. We identified four QTLs for this trait in the present study, all of which showed insignificant effects on plant height, especially those traits associated with the environment-independent locus within *Xbarc117*-*Xgwm129* on 5A. Introgression of segments containing the W7984 allele in common wheat might lengthen the uppermost internode but has no effect on plant height.

In classic genetic analysis, verification of gene-trait relationships need to be confirmed by fine mapping, map-based cloning, and transgenic analysis^42^, each step of which is laborious and time consuming. Improved annotation rates/quality of wheat genome reference assemblies will facilitate easier identification of orthologous genes of genes of known function among syntenic regions, and QTLs identified in model genomes can then be sought in crop genomes for potential inclusion in plant breeding strategies^43,44^. Moreover, the expression levels of candidate genes and/or enzyme activity and metabolite concentrations can also be used as phenotypic traits. Conditional analysis therefore provides a platform for the construction of pathway networks to bridge the gap between genes and traits of interest.

## Conclusions

In this study, we detected 4 normal QTLs and 312 conditional QTLs for plant height. In 40 QTL clusters, the genetic region of *Xbcd1970*-*Xbcd262* on chromosome 2D carried the most QTLs, with 6 normal QTLs and 39 conditional QTLs. The combination of normal and conditional QTL mapping analyses suggested that the length of the 3rd, 4th, and 5th internodes from the top had a high genetic association with plant height, whereas all photosynthesis traits showed a weaker association.

## Supplementary information

Supplementary information.
