# Supplementary material for: The contribution of photosynthesis traits and plant height components to plant height in wheat at the individual quantitative trait locus level
Source: Sci Rep. 2020 Jul 23;10:12261. doi: 10.1038/s41598-020-69138-0 (PMC7378237; doi:10.1038/s41598-020-69138-0)
Supplement: Supplementary file 1 — Supplementary information. [file 41598_2020_69138_MOESM1_ESM.docx]

Supporting information

The contribution of photosynthetic traits and plant height components toward plant height in wheat at the individual quantitative trait locus level

Ma Yu^1#^, Ze-Hou Liu^2#^, Bin Yang^1^, Hua Chen^1^, Hong Zhang^1^, Da-Bin Hou^1*^

^1^School of Life Science and Engineering, Southwest University of Science and Technology, 59 Qinglong Road, Mianyang, Sichuan, 621010, China

^2^Crop Research Institute, Sichuan Academy of Agricultural Sciences, Chengdu, 610066, Sichuan, China

^#^ Ma Yu and Ze-Hou Liu contributed equally to this research.

*** Corresponding authors:**

Da-Bin Hou, Tel: +86 816-6089523; Fax: +86 816-6089521; Email: [yuwen0073@126.com](mailto:yuwen0073@126.com)

Table S1 QTL mapping with significant LOD values for plant height and other traits

| Chrom | Interval Marker | Trait[LOD/R^2^]^a^ | | |
| --- | --- | --- | --- | --- |
|  |  | 2015-2016 | 2016-2017 | overall |
| 1A | *Xgwm136-XksuD14* | 5th\|Ci(2.63/8.26)H | PH(3.17/9.99) | PH(2.83/8.95) |
|  |  |  | PH\|Ci(3.62/11.17)S | PH\|Ci(4.45/10.61)S |
|  |  |  | PH\|Ci(3.29/9.83)H | PH\|Ci(2.71/7.56)H |
|  |  |  | PH\|CN(3/9.54)S | PH\|CN(4.89/12.05)H |
|  |  |  | PH\|CN(4.21/11.28)H | PH\|Gs(4.21/11.4)H |
|  |  |  | PH\|Gs(3.83/11.58)S | PH\|PN(3.87/9.41)S |
|  |  |  | PH\|PN(3.47/10.22)S | PH\|PN(2.9/9.47)H |
|  |  |  | PH\|PN(3.27/10.27)H | PH\|TR(3.85/10.35)S |
|  |  |  | PH\|TR(3.97/10.92)S | PH\|TR(3.02/9.12)H |
|  |  |  | PH\|TR(3.35/10.03)H | PH\|SL(2.74/9.01) |
|  |  |  | PH\|SL(3.18/10.47) | 5th\|Ci(4.23/11.39)S |
|  |  |  | PH\|IN(2.87/8.77) | 5th\|Ci(2.55/8.52)H |
|  |  |  |  | 5th\|PN(2.56/8.55)S |
|  |  |  |  | 5th\|TR(2.58/8.62)S |
|  |  |  |  | 5th\|TR(2.52/8.69)H |
| 1A | *Xcdo473-Xgwm357* |  | 2nd\|Gs(-2.9/5.78)S |  |
| 1B | *Xcdo278-Xbcd12* | SL\|Ci(-3.62/14.54)S | SL\|Ci(-2.63/9.87)S | SL\|Ci(-2.67/10.04)H |
|  |  | SL\|Ci(-2.53/10.47)H | SL\|Ci(-2.64/9.74)H | SL\|Ci(-2.79/10.83)S |
|  |  | SL\|CN(-2.65/10.86)S | SL\|Gs(-2.77/10.25)H | SL\|Gs(-2.78/10.82)H |
|  |  | SL\|Gs(-2.58/10.83)H | SL\|PN(-2.53/9.38)H | SL\|Gs(-2.57/9.7)S |
|  |  | SL\|Gs(-3.53/12.56)S | SL\|PN(-2.57/9.62)S | SL\|PN(-2.66/10.23)H |
|  |  | SL\|PN(-2.68/10.78)H |  | SL\|PN(-2.76/10.61)S |
|  |  | SL\|PN(-3.96/14.28)S |  | SL\|TR(-2.92/11.12)S |
|  |  | SL\|TR(-3.84/14.7)S |  |  |
| 1B | *Xbcd508-Xbcd1514* | CN(-2.55/8.11)H |  |  |
| 1B | *XksuI27-Xgwm140* | 1st\|CN(3.97/7.45)H | PH\|6th(3/7.55) | 1st\|CN(4.21/9.26)H |
|  |  | PH\|4th(2.79/8.95) |  | PH\|6th(3.21/7.96) |
|  |  | PH\|6th(2.96/7.19) |  |  |
|  |  | PH\|3rd(3.09/8.82) |  |  |
| 1D | *Xgwm106-Xbarc229* | 3rd\|PN(-3.45/11.81)H |  | 2nd\|PN(-2.95/12.02)H |
|  |  | 2nd\|Ci(-2.63/10.51)H |  | 2nd\|PN(-2.55/7.99)S |
|  |  | 2nd\|Gs(-2.53/10.1)H |  |  |
|  |  | 2nd\|PN(-3.16/12.62)H |  |  |
| 1D | *Xcdo89-Xbarc229* | 3rd\|Ci(-2.53/9.21)S | 3rd\|Ci(-4.02/13.82)S | 3rd\|Ci(-3.29/11.38)S |
|  |  | 3rd\|Ci(-3.06/9.59)H | 3rd\|Ci(-4.22/14.36)H | 3rd\|Ci(-3.79/12.78)H |
|  |  | 3rd\|CN(-2.89/9.15)S | 3rd\|CN(-2.98/9.77)H | 3rd\|Gs(-4.13/13.63)H |
|  |  | 3rd\|PN(-2.86/10.56)S | 3rd\|CN(-3.22/11.17)S | 3rd\|CN(-3.58/10.35)S |
|  |  | 3rd\|TR(-2.75/9.73)H | 3rd\|Gs(-4.72/15.19)S | 3rd\|Gs(-3.56/11.79)H |
|  |  | 3rd\|TR(-3.21/10.07)S | 3rd\|PN(-3.34/11.23)H | 3rd\|Gs(-4.01/12.74)S |
|  |  | 3rd(-3.25/10.3) | 3rd\|PN(-4.14/14.52)S | 3rd\|PN(-2.97/9.96)H |
|  |  |  | 3rd\|TR(-4.1/14.07)H | 3rd\|PN(-3.57/12.78)S |
|  |  |  | 3rd\|TR(-4.28/14.84)S | 3rd\|TR(-3.42/11.85)H |
|  |  |  | 3rd(-4.61/15.74) | 3rd\|TR(-4.15/12.39)S |
|  |  |  |  | 3rd(-4/13.62) |
| 2A | *Xgwm210-Xbarc1138* | 3rd(2.58/8.38) | 2nd\|Gs(3.7/7.07)S | 2nd\|CN(2.91/9.4)S |
|  |  | 3rd\|CN(2.77/9.03)S |  | 2nd\|Gs(3.38/7.41)S |
|  |  | 3rd\|Ci(2.58/8.39)H |  | 2nd\|Ci(3.59/7.68)H |
|  |  |  |  | 3rd\|CN(3.1/9.22)S |
| 2A | *Xfba178-Xfbb329* |  | 6th\|CN(-2.54/8.93)H |  |
|  |  |  | 6th\|CN(-2.82/8.98)S |  |
| 2A | *Xgwm294-Xbcd161* |  | 2nd\|Gs(2.92/7.21)S | 2nd\|Gs(3.25/8.73)S |
|  |  |  |  | 2nd\|Ci(3.33/8.75)H |
| 2A | *Xgwm445-Xbarc353* | PH\|5th(3.38/13.17) | PH\|5th(5.19/17.12) | PH\|5th(3.59/14.9) |
|  |  | 2nd\|TR(2.61/11.26)S | 2nd\|Ci(2.7/11.38)S | 2nd\|CN(3.69/14.51)S |
|  |  |  | 2nd\|Ci(3.01/12.59)H | 2nd\|PN(3.58/14.07)S |
|  |  |  | 2nd\|Gs(3.17/12.93)H | 2nd\|TR(2.68/11.49)H |
|  |  |  | 2nd\|TR(2.72/11.43)S | 2nd\|TR(3.38/13.2)S |
|  |  |  | 2nd\|TR(3.61/12)H |  |
|  |  |  | 2nd(2.74/10.7) |  |
| 2A | *XksuF11-Xfba314* | IN\|Ci(-2.81/9.55)H | IN\|Ci(-2.78/9.6)H | IN\|Ci(-2.61/8.88)H |
|  |  | IN\|Ci(-2.97/11.12)S | IN\|Gs(-2.82/9.72)S | IN\|CN(-2.9/9.66)S |
|  |  | IN\|CN(-2.88/9.9)S | IN\|PN(-2.7/9.38)S | IN\|CN(-3.92/11.84)H |
|  |  | IN\|CN(-7.24/16.87)H |  | IN\|Gs(-2.54/8.69)H |
|  |  | IN\|Gs(-2.92/10.51)S |  | IN\|Gs(-2.69/9.2)S |
|  |  | IN\|Gs(-3.08/10.66)H |  | IN\|PN(-2.84/9.65)S |
|  |  | IN\|PN(-2.91/10.41)S |  | IN\|TR(-2.66/9.18)S |
|  |  | IN\|TR(-2.89/10.6)S |  | IN\|TR(-2.8/9.79)H |
|  |  | IN(-2.95/10.13) |  | IN(-2.77/9.4) |
| 2B | *Xbarc361-Xgwm388* | 1st\|Ci(3.34/11.51)S | 1st\|CN(2.75/11.1)H | 1st\|Ci(3.19/11.06)S |
|  |  | 1st\|Ci(3.63/11.1)H | PH\|5th(3.82/9.86) | 1st\|CN(3.77/8.24)H |
|  |  | 1st\|CN(3.45/6.68)H | PH\|IN(2.87/9.1) | 1st(3.51/8.51) |
|  |  | 1st\|Gs(3.3/10.88)S | PH\|6th(4.23/11.21) | PH\|5th(3.13/10.1) |
|  |  | 1st\|PN(4.06/9.01)S |  | PH\|IN(2.83/9.31) |
|  |  | 1st\|TR(3.42/8.06)S |  | PH\|6th(4.34/11.31) |
|  |  | 1st(3.51/8.38) |  |  |
|  |  | PH\|5th(3.14/9.24) |  |  |
|  |  | PH\|IN(3.19/10.64) |  |  |
| 2B | *Xcdo678-Xmwg660* | CN(3.94/10.13)S |  |  |
| 2D | *Xbcd1970-Xbcd262* | 5th\|Ci(2.98/10.74)S | 5th\|Ci(3.67/14.59)S | 5th\|Ci(3.99/14.23)H |
|  |  | 5th\|Ci(4.22/13)H | 5th\|Ci(4.1/16.14)H | 5th\|Ci(4.95/12.76)S |
|  |  | 5th\|CN(2.97/10.86)H | 5th\|CN(2.86/10.81)S | 5th\|CN(3.39/12.14)H |
|  |  | 5th\|CN(4.02/14.42)S | 5th\|CN(3.59/14.56)H | 5th\|CN(4.17/15.01)S |
|  |  | 5th\|Gs(3.1/12.06)S | 5th\|Gs(3.98/15.7)H | 5th\|Gs(3.83/14.41)S |
|  |  | 5th\|Gs(3.68/13.55)H | 5th\|Gs(3.99/15.7)S | 5th\|Gs(4.11/15.42)H |
|  |  | 5th\|PN(3.22/12.54)S | 5th\|PN(3.76/14.86)H | 5th\|PN(3.91/14.67)H |
|  |  | 5th\|PN(3.35/12.46)H | 5th\|PN(4.12/16.19)S | 5th\|PN(3.94/14.05)S |
|  |  | 5th\|TR(3.15/12.29)S | 5th\|TR(3.95/15.84)H | 5th\|TR(3.19/11.56)H |
|  |  | 5th(3.2/11.98) | 5th\|TR(4.07/16.01)S | 5th\|TR(3.92/13.95)S |
|  |  | IN\|Ci(4.36/16.8)S | 5th(3.92/15.38) | 5th(3.66/13.82) |
|  |  | IN\|Ci(5.7/20.58)H | IN\|Ci(4.75/18.6)S | IN\|Ci(4.91/19.13)S |
|  |  | IN\|CN(5.13/11.37)H | IN\|Ci(5.18/18.71)H | IN\|Ci(5.7/20.71)H |
|  |  | IN\|CN(5.91/21.23)S | IN\|CN(4.44/17.4)H | IN\|CN(4.92/15.18)H |
|  |  | IN\|Gs(5.05/18.14)H | IN\|CN(4.65/18.33)S | IN\|CN(6.03/21.48)S |
|  |  | IN\|Gs(5.46/20.76)S | IN\|Gs(4.87/18.9)H | IN\|Gs(5.43/19.72)H |
|  |  | IN\|PN(4.81/18.94)H | IN\|Gs(5.15/18.6)S | IN\|Gs(5.54/20.13)S |
|  |  | IN\|PN(5.43/20.56)S | IN\|PN(4.03/15.96)H | IN\|PN(4.77/18.79)H |
|  |  | IN\|TR(3.96/15.52)H | IN\|PN(5.16/18.78)S | IN\|PN(5.61/20.2)S |
|  |  | IN\|TR(4.96/18.98)S | IN\|TR(4.98/19.3)H | IN\|TR(4.83/17.59)H |
|  |  | IN(5.42/19.58) | IN\|TR(5.08/19.68)S | IN\|TR(5.25/19.15)S |
|  |  | PH\|2nd(4.61/11.18) | IN(5.38/20.74) | IN(5.61/20.25) |
|  |  | 2nd\|CN(-3.12/13.26)S | PH\|2nd(5/15.65) | PH\|2nd(7.27/14.63) |
|  |  | 2nd\|Gs(-5.13/13.07)S | 2nd\|Ci(-3.03/12.1)H | 2nd\|Ci(-2.66/12.55)S |
|  |  | 2nd(-3.4/13.36) | 2nd\|Ci(-3/11.9)S | 2nd\|Ci(-4.86/12.13)H |
|  |  | 6th\|Ci(5.15/21.09)S | 2nd\|CN(-3.26/11.79)H | 2nd\|CN(-2.64/8.84)S |
|  |  | 6th\|Ci(7.22/27)H | 2nd\|CN(-4.3/15.69)S | 2nd\|CN(-2.91/9.28)H |
|  |  | 6th\|CN(10.6/28.04)S | 2nd\|Gs(-3/11.61)H | 2nd\|Gs(-3.15/12.72)H |
|  |  | 6th\|CN(8.97/26.41)H | 2nd\|Gs(-7.16/16.8)S | 2nd\|Gs(-5.13/13.07)S |
|  |  | 6th\|Gs(7.13/26.3)H | 2nd\|PN(-2.65/10.6)H | 2nd\|TR(-2.6/8.68)S |
|  |  | 6th\|Gs(7.27/26.05)S | 2nd\|PN(-4.45/15.45)S | 2nd(-3.79/14.41) |
|  |  | 6th\|PN(6.72/24.75)H | 2nd\|TR(-2.7/10.5)S | 6th\|Ci(6.93/25.6)S |
|  |  | 6th\|PN(7/25.12)S | 2nd(-3.53/13.42) | 6th\|Ci(7.42/27.73)H |
|  |  | 6th\|TR(4.23/16.84)H | 6th\|Ci(6.77/24.68)S | 6th\|CN(6.82/25.52)H |
|  |  | 6th\|TR(6.47/23.18)S | 6th\|Ci(6.92/26.19)H | 6th\|CN(8.62/29.06)S |
|  |  | 6th(7.16/26.61) | 6th\|CN(6.26/21.83)H | 6th\|Gs(7.45/27.58)H |
|  |  | TR(3.55/12.7)H | 6th\|CN(7.27/22.89)S | 6th\|Gs(7.47/27.86)S |
|  |  |  | 6th\|Gs(6.38/24.13)H | 6th\|PN(6.88/25.36)H |
|  |  |  | 6th\|Gs(7.02/26.44)S | 6th\|PN(7.42/27.56)S |
|  |  |  | 6th\|PN(5.57/20.98)H | 6th\|TR(6.87/25.46)H |
|  |  |  | 6th\|PN(6.75/25.54)S | 6th\|TR(7.08/26.34)S |
|  |  |  | 6th\|TR(6.77/25.32)S | 6th(7.49/27.71) |
|  |  |  | 6th\|TR(7.28/25.13)H | TR(2.79/11.34)H |
|  |  |  | 6th\|TR(7.08/26.34)S |  |
|  |  |  | 6th(7.09/26.46) |  |
|  |  |  | TR(2.88/7.17)S |  |
| 2D | *Xbcd262-Xwsu1* | 1st\|TR(-2.99/4.88)H | 1st\|PN(-2.62/4.63)S | 1st\|CN(-2.77/4.72)S |
|  |  | 1st\|CN(-3.08/6.51)S | 1st\|CN(-2.56/5.87)S | 1st\|PN(-2.82/4.76)S |
|  |  | 1st\|PN(-2.73/5.82)H | 1st\|Ci(-2.62/6.66)H | 1st\|TR(-3.37/6.3)S |
|  |  |  |  | 1st\|Ci(-2.88/4.83)H |
|  |  |  |  | 1st\|TR(-2.66/4.47)H |
| 2D | *Xgwm666-Xmwg30* | 1st\|Ci(-2.53/7.82)H | 1st\|Ci(-2.66/8.18)H | 1st\|CN(-3.37/8.98)H |
|  |  | 1st\|CN(-4.66/10.71)S |  | 1st(-2.91/8.59) |
|  |  | 1st\|Gs(-3.59/9.55)H |  |  |
|  |  | 1st(-3.16/9.27) |  |  |
|  |  | Gs(-5.7/12.49)S |  |  |
| 3B | *Xfba311-Xbarc75* | PH\|Ci(3.62/11.22)S | PH\|Ci(2.5/8.02)S | PH\|2nd(6.09/12.24) |
|  |  | PH\|CN(3.12/9.69)S | PH\|Ci(2.72/8.51)H | PH\|TR(2.59/8.2)H |
|  |  | PH\|Gs(3.18/8.66)H | PH\|Gs(2.78/8.76)S | PH\|TR(2.91/8.2)S |
|  |  | PH\|Gs(3.96/12.05)S | PH\|Gs(2.85/8.2)H | 5th\|Ci(2.71/6.58)S |
|  |  | PH\|PN(3.54/10.88)S | PH\|2nd(2.83/8.9) |  |
|  |  | PH\|2nd(4.88/12.06) | PH\|TR(2.57/8.05)H |  |
|  |  | PH\|TR(2.54/8.12)H |  |  |
|  |  | PH\|TR(2.94/7.52)S |  |  |
|  |  | 6th\|CN(3.66/9.48)S |  |  |
|  |  | Gs(3.26/6.39)S |  |  |
| 3B | *Xbarc133-Xbarc147* | 6th\|CN(3/7.46)H |  |  |
| 3B | *Xfba133-Xfba360* | PH\|1st(2.6/4.95) |  |  |
| 3B | *Xfba8Hb-Xfba167* | 6th\|PN(-2.66/8.61)S |  |  |
|  |  | 6th\|Gs(-2.57/8.21)S |  |  |
| 3D | *XksuA6-Xgwm2* |  | 2nd\|Gs(-4.92/9.87)S | 2nd\|Gs(-4.12/9.28)S |
|  |  |  |  | 2nd\|Ci(-4.35/9.6)H |
| 3D | *Xgwm341-Xbarc6* | SL\|TR(-2.59/5.68)H |  |  |
| 3D | *Xwg110-Xgwm3* |  | CN(3.86/12.57)H | CN(2.85/11.4)H |
| 4A | *Xfbb1-Xfba40* | 1st\|CN(-3.95/7.83)S | 1st\|CN(-3.84/9.18)S | 1st\|CN(-4.51/8.85)S |
|  |  | 1st\|CN(-2.97/5.84)H | 1st\|PN(-4.43/9.05)S | 1st\|PN(-4.63/9)S |
|  |  | 1st\|PN(-4.58/9.46)H | 1st\|PN(-4.43/10.35)H | 1st\|PN(-4.71/10.43)H |
|  |  | 1st\|Gs(-3.01/6.77)H | 1st\|Gs(-3.82/9.3)H | 1st\|Ci(-4.78/9.25)H |
|  |  | 1st\|TR(-5.04/9.44)H | 1st\|Ci(-3.21/8.58)H | 1st\|TR(-4.86/9.47)H |
|  |  |  | 1st\|TR(-4.04/9.74)H | 1st\|TR(-5.05/10.01)S |
|  |  |  | PH\|3rd(-2.68/9.62) |  |
| 4A | *Xgwm4-Xgwm192* | SL(-3.37/16.6) | SL(-5.53/16.14) | SL(-4.06/18.98) |
|  |  | SL\|CN(-3.41/15.16)S | SL\|CN(-4.93/21.69)S | SL\|CN(-4.26/19.72)S |
|  |  | SL\|CN(-3.43/14.37)H | SL\|CN(-5.29/21.38)H | SL\|CN(-3.82/17.63)H |
|  |  | SL\|PN(-4.29/16.53)S | SL\|PN(-5.06/21.15)S | SL\|PN(-4.47/18.95)S |
|  |  | SL\|Gs(-4.51/17.54)S | SL\|Gs(-5.34/22.82)S | SL\|Gs(-4.75/19.98)S |
|  |  | SL\|Ci(-3.83/16.49)S | SL\|Ci(-5.1/21.28)S | SL\|Ci(-4.38/18.62)S |
|  |  | SL\|TR(-4.31/17.68)S | SL\|TR(-5.26/16.72)S | SL\|TR(-4.58/19.02)S |
|  |  | SL\|PN(-3.66/15.94)H | SL\|PN(-5.15/21.4)H | SL\|PN(-4.49/19.01)H |
|  |  | SL\|Gs(-3.19/14.32)H | SL\|Gs(-4.93/20.31)H | SL\|Gs(-4.18/17.76)H |
|  |  | SL\|Ci(-3.48/15.69)H | SL\|Ci(-5.04/20.78)H | SL\|Ci(-4.55/18.88)H |
|  |  |  | SL\|TR(-5.33/23.56)H | SL\|TR(-5.8/22.07)H |
| 4A | *Xbcd402-XksuG12* | IN\|CN(3.28/7.53)H |  |  |
| 4A | *Xgwm397-Xfba4* | SL\|TR(-7.58/22.09)H | 4th\|Ci(-3.63/7.29)S |  |
|  |  |  | 4th\|TR(-3.44/6.85)H |  |
| 4A | *XksuD9-Xfbb154* | 1st\|PN(-3.99/8.56)S |  | 2nd\|CN(-3.85/12.4)H |
|  |  | 1st\|TR(-3.98/9.08)S |  |  |
|  |  | 2nd\|CN(-3.33/11.06)H |  |  |
| 4A | *XksuE3-Xfba282* |  | SL(-4.03/11.92) |  |
|  |  |  | 2nd\|CN(-3.12/11.41)H |  |
|  |  |  | SL\|TR(-3.85/12.73)S |  |
|  |  |  | 2nd(-2.59/8.29) |  |
| 4B | *Xfba177-Xbcd402* |  | SL\|TR(2.52/7.39)S |  |
|  |  |  | SL(2.94/7.99) |  |
| 4D | *Xbarc308-Xbarc217* | CN(3.1/10.08)S |  |  |
| 4D | *Xfbb178-Xbcd1431* |  |  |  |
|  |  | PH\|1st(-6.17/13.14) |  |  |
| 4D | *Xfbb226-Xcdo949* | 3rd\|Gs(-2.75/10.28)H | CN(5.25/17.12)H | CN(4.1/15.84)H |
|  |  | CN(3.18/10.45)H |  |  |
|  |  | 6th\|TR(-2.78/10.82)H |  |  |
| 5A | *Xbarc117-Xgwm129* | 1st(6.61/17.1) | 1st(4.37/16.27) | 1st(6.47/17.02) |
|  |  | 1st\|CN(8.63/17.76)S | 1st\|CN(6.68/16.06)S | 1st\|CN(9.02/18.41)S |
|  |  | 1st\|CN(8.67/18.37)H | 1st\|CN(3.27/13.83)H | 1st\|CN(6.43/15.16)H |
|  |  | 1st\|PN(7.41/17.49)S | 1st\|PN(8.48/17.8)S | 1st\|PN(9.18/18.55)S |
|  |  | 1st\|Gs(5.13/17.46)S | 1st\|Gs(3.6/16.73)S | 1st\|Gs(3.69/17.83)S |
|  |  | 1st\|Ci(5.1/17.92)S | 1st\|Ci(3.31/16.36)S | 1st\|Ci(4.51/15.42)S |
|  |  | 1st\|TR(5.61/13.57)S | 1st\|TR(3.42/16.08)S | 1st\|TR(9.41/21.01)S |
|  |  | 1st\|PN(7.92/17.05)H | 1st\|PN(7.35/17.33)H | 1st\|PN(7.93/18.04)H |
|  |  | 1st\|Gs(7.17/16.95)H | 1st\|Gs(6.56/15.86)H | 1st\|Gs(3.8/17.03)H |
|  |  | 1st\|Ci(5.2/15.71)H | 1st\|Ci(6.26/16.72)H | 1st\|Ci(9.42/19.05)H |
|  |  | 1st\|TR(9.56/18.81)H | 1st\|TR(6.57/15.9)H | 1st\|TR(9.54/19.45)H |
|  |  | PH\|4th(3.97/12.81) | PH\|3rd(5.04/19.21) | PH\|3rd(5.28/17.27) |
|  |  | PH\|3rd(5.99/18.29) |  |  |
| 5A | *Xgwm293-Xcdo785* | PH\|6th(5.53/16.12) | PH\|6th(5.36/16.48) | PH\|6th(5.25/15.77) |
|  |  | PH\|SL(3.25/12.96) | PH(4.56/15.19) | PH(4.64/15.63) |
|  |  | PH\|IN(4.22/14.39) | PH\|SL(3.63/12.3) | PH\|SL(3.87/13.31) |
|  |  |  | PH\|IN(5.03/16.46) | PH\|IN(4.18/13.98) |
| 5A | *Xgwm186-Xbcd1949* | 3rd(3.64/12.05) | 3rd(3.46/12.24) | 3rd(3.3/11.78) |
|  |  | 4th\|CN(4.14/16.3)H | 4th\|CN(3.69/14.57)H | 4th\|CN(4/16.09)S |
|  |  | 3rd\|CN(4.57/15.26)S | 3rd\|CN(4.21/15.48)S | 3rd\|CN(5.88/18.4)S |
|  |  | 3rd\|CN(4.67/17.88)H | 3rd\|CN(5.28/19.1)H | 3rd\|CN(5.14/18.8)H |
|  |  | 3rd\|PN(3.88/15.6)S | 3rd\|PN(3.69/13.51)S | 4th\|Gs(3.2/12.94)S |
|  |  | 3rd\|Gs(4.94/15.54)S | 3rd\|Gs(4.11/13.63)S | 3rd\|PN(3.53/13.34)S |
|  |  | 3rd\|Ci(3.65/14.62)S | 3rd\|Ci(4.15/15.1)S | 3rd\|Gs(4.32/14.48)S |
|  |  | 3rd\|TR(4.89/16.86)S | 3rd\|TR(3.94/14.33)S | 3rd\|Ci(3.97/14.83)S |
|  |  | 3rd\|PN(4.84/14.37)H | 4th\|Gs(2.93/11.48)H | 3rd\|TR(5/15.96)S |
|  |  | 3rd\|Gs(3.22/12.67)H | 3rd\|PN(4.27/15.29)H | 4th\|Gs(3.3/13)H |
|  |  | 3rd\|Ci(4.08/13.58)H | 3rd\|Gs(4.12/14.5)H | 3rd\|PN(4.37/15.88)H |
|  |  | 3rd\|TR(3.83/14.8)H | 3rd\|Ci(4.06/14.62)H | 3rd\|Gs(4.3/15.34)H |
|  |  |  | 3rd\|TR(3.99/14.5)H | 3rd\|Ci(3.96/14.26)H |
|  |  |  |  | 3rd\|TR(4.01/14.96)H |
| 5A | *Xmwg522-Xbarc330* | PH(5.39/17.28) | PH\|Ci(4.83/16.09)S | PH\|Ci(6.31/16.26)S |
|  |  | PH\|Ci(6.01/19.56)H | PH\|Ci(5.25/17.12)H | PH\|Ci(6.37/19.71)H |
|  |  | PH\|Ci(7.34/23.69)S | PH\|CN(5.29/17.95)S | PH\|CN(4.82/18.5)S |
|  |  | PH\|CN(5.87/18.1)S | PH\|CN(6.44/18.31)H | PH\|CN(6.88/17.78)H |
|  |  | PH\|CN(6.65/21.93)H | PH\|1st(3.52/13.79) | PH\|1st(3.58/14.07) |
|  |  | PH\|1st(6.39/13.86) | PH\|Gs(5.04/13.94)H | PH\|Gs(5.44/17.9)S |
|  |  | PH\|Gs(6.46/18.1)H | PH\|Gs(5.23/16.92)S | PH\|Gs(5.62/20.29)H |
|  |  | PH\|Gs(6.97/21.76)S | PH\|PN(4.5/13.72)S | PH\|PN(4.84/16.58)H |
|  |  | PH\|PN(5.54/18.64)H | PH\|PN(4.89/16.07)H | PH\|PN(6.31/16.58)S |
|  |  | PH\|PN(7.32/23.31)S | PH\|2nd(3.15/9.65) | PH\|2nd(7.91/16.33) |
|  |  | PH\|2nd(5.9/14.08) | PH\|TR(4.72/13.28)S | PH\|TR(5.65/15.74)S |
|  |  | PH\|TR(6.08/19.98)H | PH\|TR(5.52/18.29)H | PH\|TR(5.76/19.28)H |
|  |  | PH\|TR(8.96/25.18)S | 5th\|CN(2.61/10.61)S | 5th\|Ci(2.74/9.71)H |
|  |  | 5th\|Ci(3.69/13.3)S | 4th\|Ci(4.35/15.15)H | 5th\|Ci(3.61/9.15)S |
|  |  | 5th\|Ci(3.88/12.01)H | 4th\|Ci(4.44/9.36)S | 5th\|CN(3.08/12.21)H |
|  |  | 5th\|CN(3.79/13.77)S | 4th\|CN(3.43/13.3)S | 5th\|CN(3.7/13.43)S |
|  |  | 5th\|CN(3.8/14.43)H | 4th\|Gs(4.47/11.43)S | 5th\|Gs(2.61/9.65)H |
|  |  | 5th\|Gs(3.11/12.34)S | 4th\|PN(4.59/11.66)S | 5th\|Gs(2.74/10.27)S |
|  |  | 5th\|Gs(3.26/12.16)H | 4th\|TR(4.62/9.72)H | 5th\|PN(2.75/10.24)H |
|  |  | 5th\|PN(3.14/12.44)S | 4th\|TR(4.81/12.09)S | 5th\|PN(2.82/10)S |
|  |  | 5th\|PN(3.18/12.04)H | 4th(3.67/11.21) | 5th\|TR(2.83/10.03)S |
|  |  | 5th\|TR(3.07/12.18)S |  | 5th\|TR(2.84/10.45)H |
|  |  | 5th\|TR(3.08/12.23)H |  | 5th(2.6/9.79) |
|  |  | 5th(3.04/11.55) |  | 4th\|Ci(4.66/16.23)H |
|  |  | 4th\|Ci(3.66/14.74)H |  | 4th\|Ci(4.68/16.24)S |
|  |  | 4th\|Ci(4.29/17.4)S |  | 4th\|CN(4.11/15.85)H |
|  |  | 4th\|CN(3.99/15.88)S |  | 4th\|PN(3.5/13.94)H |
|  |  | 4th\|Gs(3.65/15.27)H |  | 4th\|PN(4.59/15.93)S |
|  |  | 4th\|Gs(3.98/16.1)S |  | 4th\|TR(3.46/13.65)S |
|  |  | 4th\|PN(3.78/15.06)H |  | 4th\|TR(4.77/16.67)H |
|  |  | 4th\|PN(4.1/16.79)S |  | 4th(4.23/14.37) |
|  |  | 4th\|TR(3.49/13.91)H |  |  |
|  |  | 4th\|TR(4.14/16.55)S |  |  |
|  |  | 4th(3.05/12.32) |  |  |
|  |  | 6th\|CN(3.26/8.49)H |  |  |
|  |  |  |  |  |
| 5A | *Xgwm666-Xrz395* |  | 2nd\|Gs(3.2/6.18)S | 2nd\|Gs(3.14/7.72)S |
|  |  |  |  | 2nd\|Ci(3.84/9.2)H |
|  |  |  |  | 1st\|TR(2.52/4.78)S |
| 5A | *Xfba68-Xfbb209* | SL\|TR(3.62/8.07)H | PN(-3.06/12.35)S |  |
| 5B | *Xfba127-Xbcd1140* | PH\|Ci(2.54/8.36)S |  | PH\|6th(2.51/6.75) |
|  |  | PH\|CN(2.81/8.93)S |  | PH\|2nd(3.62/6.91) |
|  |  | PH\|Gs(3.14/8.62)H |  | PH\|IN(2.81/10.42) |
|  |  | PH\|TR(3.21/10.75)H |  |  |
|  |  | PH\|TR(3.49/9.46)S |  |  |
|  |  | PH\|5th(2.83/8.99) |  |  |
| 5B | *Xabg473-Xmwg914* | 1st\|PN(3.15/6.63)S |  | PN(-2.65/9.41)H |
|  |  | 1st\|TR(3.12/7.01)S |  |  |
|  |  | PN(-2.63/8.72)H |  |  |
| 5B | *Xfbb237-Xbcd1030* | 1st(-3.55/8.38) |  | 1st(-3.51/8.48) |
|  |  | 1st\|TR(-4.89/12.45)S |  | 1st\|Ci(-2.77/8.82)S |
|  |  | 1st\|Ci(-3.05/10.29)S |  |  |
|  |  | 1st\|Ci(-3.15/8.79)H |  |  |
|  |  | 1st\|Gs(-3.85/12.71)S |  |  |
|  |  | 1st\|PN(-5.85/12.83)S |  |  |
| 5D | *Xfbb156-Xcdo57* |  | PN(3.33/12.18)H | PN(3.68/12.63)H |
| 5D | *Xmwg900-Xbarc322* | Gs(-4.47/10.01)S |  |  |
| 6A | *Xcmwg652-Xfba85* |  | 4th(-2.83/8.38) | CN(-3.5/14.92)S |
|  |  |  | CN(-2.89/14.08)S |  |
|  |  |  | 4th\|Ci(-5.03/10.26)S |  |
|  |  |  | 4th\|Gs(-3.32/8.05)S |  |
|  |  |  | 4th\|PN(-3.44/8.33)S |  |
|  |  |  | 4th\|TR(-3.44/8.14)S |  |
|  |  |  | 4th\|TR(-4.99/10.14)H | |
| 6A | *Xcdo29-Xtam36* | PH(3.13/9.33) | 4th\|Ci(2.65/8.42)H | 4th\|Ci(2.68/8.57)H |
|  |  | PH\|Ci(2.95/8.78)H | 4th\|Ci(4.67/9.29)S | 4th\|Ci(2.73/8.72)S |
|  |  | PH\|Ci(3.07/8.48)H | 4th\|Gs(4.55/11.09)S | 4th\|PN(2.67/8.53)S |
|  |  | PH\|CN(2.91/8.62)H | 4th\|PN(4.78/11.7)S | 4th\|TR(2.56/8.16)H |
|  |  | PH\|1st(3.49/6.75) | 4th\|TR(4.65/9.22)H | 4th(2.91/9.2) |
|  |  | PH\|PN(2.68/8.28)H | 4th\|TR(5.15/12.41)S | PH\|Ci(3.07/8.48)H |
|  |  | TR(3.69/12.08)H | 4th(3.85/11.19) | PH\|Ci(4.06/9.52)S |
|  |  |  |  | PH\|Gs(2.53/8.42)H |
|  |  |  |  | PH\|Gs(2.99/9.09)S |
|  |  |  |  | PH\|PN(3.81/9.15)S |
|  |  |  |  | TR(2.66/9.53)H |
| 6A | *Xfbb170-Xcdo388* |  | PH\|2nd(3.52/10.19) |  |
| 6A | *Xcsb112(Dhn5)-Xfba111* |  | PH\|PN(2.73/9.92)S |  |
| 6B | *Xmwg74-Xfba251* | 2nd\|TR(-2.51/8.87)S | 3rd\|Gs(-2.61/8.11)S | 3rd(-2.5/8.12) |
|  |  | 3rd\|TR(-2.94/9.35)S |  | 3rd\|TR(-2.69/8.27)S |
|  |  |  |  | 3rd\|Ci(-2.59/8.31)H |
|  |  |  |  | 3rd\|Gs(-2.83/9.14)S |
| 6D | *Xfbb231-Xpsr106* | SL\|TR(-3.51/7.5)H |  |  |
| 6D | *Xbarc202-Xbarc123* |  |  | CN(3.11/12.4)S |
| 7A | *Xcdo545-Xgwm666* |  | TR(-3.83/9.63)S |  |
| 7A | *Xfba127-Xfba109* | PH\|Gs(2.78/9.02)H | PH\|5th(2.82/7.71) | PH\|2nd(2.88/5.2) |
|  |  |  | PH\|Ci(2.59/7.53)H | PH\|TR(2.71/7.87)S |
|  |  |  | PH\|Gs(3.16/9.39)H |  |
|  |  |  | PH\|PN(2.65/8.25)H |  |
|  |  |  | PH\|TR(2.81/7.77)S |  |
| 7A | *Xfbb186-Xcdo475* | SL\|CN(2.88/10.83)H |  |  |
|  |  | SL\|PN(2.58/8.81)S |  |  |
|  |  | SL\|Gs(2.61/8.93)S |  |  |
|  |  | SL\|TR(3.86/8.32)H |  |  |
|  |  | PH\|2nd(2.85/6.27) |  |  |
| 7A | *Xcdo347-Xfba134* |  |  | Gs(2.75/10.56)H |
| 7B | *Xfba42-Xgwm68* | IN\|CN(2.58/5.62)H | Ci(6.2/18.13)S | Ci(3.78/14.49)S |
|  |  |  | TR(4.85/14.43)S | IN\|CN(2.69/8.2)H |
| 7B | *Xrz476-Xcnl7* | PH\|1st(-4.95/9.83) |  | PH\|CN(-2.65/6.49)H |
|  |  |  |  | PH\|PN(-2.59/6.17)S |
|  |  |  |  | PH\|Ci(-2.97/6.89)S |
| 7B | *XksuE18-Xcnl2* | CN(-2.77/7.46)S |  |  |
| 7D | *Xwg834-Xbarc154* | Ci(4.47/18.17)S |  |  |
|  |  | Gs(-2.64/5.2)S |  |  |
| 7D | *Xwg420-Xfba204* | 3rd\|Gs(3.59/12.07)S |  |  |

^a^ Normal traits are plant height (PH); spike length (SL); internode number (IN); length of first internode from the top (1st); length of second internode from the top (2nd); length of third internode from the top (3rd); length of fourth internode from the top (4th); length of fifth internode from the top (5th); length of sixth internode from the top (6th); chlorophyll content (CN); net photosynthetic rate (PN); stomatal conductance (Gs); intercellular CO2 concentration at (Ci); transpiration rate (TR); respectively. Conditional trait A|B is trait A that have removed the influences of trait B. Numerals before parentheses are LOD peak of the QTL. E and numerals in parentheses indicate the environment in which the QTL was detected and the percentage of phenotypic variance explained by the additive effects of the mapped QTL, respectively. Negative signs indicate that ‘W7984’ alleles reduce phenotypic value whereas positive indicate that ‘W7984’ alleles increase the phenotypic value. Words of S and H after parentheses indicate this QTL was identified at seedling stage and heading stage, respectively.
